# New Insights into the Intrinsic and Extrinsic Factors That Shape the Human Skin Microbiome

**DOI:** 10.1128/mBio.00839-19

**Published:** 2019-07-02

**Authors:** Pedro A. Dimitriu, Brandon Iker, Kausar Malik, Hilary Leung, W. W. Mohn, Greg G. Hillebrand

**Affiliations:** a Microbiome Insights, Inc., Vancouver, British Columbia, Canada; b Department of Microbiology and Immunology, Life Sciences Institute, University of British Columbia, Vancouver, British Columbia, Canada; c Amway Corporation, Ada, Michigan, USA; University of Toronto; University of Pennsylvania; City University of Hong Kong

**Keywords:** *Corynebacterium*, age, demographic, forehead, host lifestyle, host physiology, metadata, scalp, skin microbiome

## Abstract

Despite recognition that biogeography and individuality shape the function and composition of the human skin microbiome, we know little about how extrinsic and intrinsic host factors influence its composition. To explore the contributions of these factors to skin microbiome variation, we profiled the bacterial microbiomes of 495 North American subjects (ages, 9 to 78 years) at four skin surfaces plus the oral epithelium using 16S rRNA gene amplicon sequencing. We collected subject metadata, including host physiological parameters, through standardized questionnaires and noninvasive biophysical methods. Using a combination of statistical modeling tools, we found that demographic, lifestyle, and physiological factors collectively explained 12 to 20% of the variability in microbiome composition. The influence of health factors was strongest on the oral microbiome. Associations between host factors and the skin microbiome were generally dominated by operational taxonomic units (OTUs) affiliated with the *Clostridiales* and *Prevotella*. A subset of the correlations between microbial features and host attributes were site specific. To further explore the relationship between age and the skin microbiome of the forehead, we trained a Random Forest regression model to predict chronological age from microbial features. Age was associated mostly with two mutually coexcluding *Corynebacterium* OTUs. Furthermore, skin aging variables (wrinkles and hyperpigmented spots) were independently correlated to these taxa.

## Abstract (precis)

Many studies have highlighted the importance of body site and individuality in shaping the composition of the human skin microbiome, but we still have a poor understanding of how extrinsic (e.g., lifestyle) and intrinsic (e.g., age) factors influence its composition. We characterized the bacterial microbiomes of North American volunteers at four skin sites and the mouth. We also collected extensive subject metadata and measured several host physiological parameters. Integration of host and microbial features showed that the skin microbiome was predominantly associated with demographic, lifestyle, and physiological factors. Furthermore, we uncovered reproducible associations between chronological age, skin aging, and members of the genus *Corynebacterium*. Our work provides new understanding of the role of host selection and lifestyle in shaping skin microbiome composition. It also contributes to a more comprehensive appreciation of the factors that drive interindividual skin microbiome variation.

## INTRODUCTION

The skin is the largest epithelial interface separating the human body from the outside environment, and its surface is colonized by a diverse community of bacteria, fungi, and viruses (1–3). These commensal microorganisms play critical roles in lipid metabolism, colonization resistance to transient organisms, and regulation of the immune system (4). Available evidence indicates strong body site specificity and individuality of community composition and functional potential, with variation of physiological conditions driving the local microbial communities. For instance, oily surfaces such as the forehead support lipophilic bacteria, whereas dry sites such as the forearm harbor a more diverse community (3, 5–7). Like the distribution of skin microbes, the temporal variability of the skin microbiome is site specific (8). These observations suggest that skin microbiomes are primarily structured by host selection pressures (e.g., local physiological conditions). However, we have limited quantitative understanding of how demographic and anthropometric variability, two intrinsic components of human populations, influence skin microbiome composition.

In addition to host physiology, extrinsic perturbations also likely shape the skin microbiome. Skin microbes are thought to remain stable once intrinsic factors, such as the period leading to adolescence, stabilize (8). However, it is possible that age-independent extrinsic factors, including medications and lifestyle, are associated with shifts in the skin microbiome. It is well known that older people are frequently predisposed to the development of inflammatory conditions (9), and this may facilitate colonization by specific microbial taxa. In addition, the skin’s chemical landscape can be strongly impacted by skin care products and cleansers (10): certain compounds present in these products may also contribute to skin microbiome variation, particularly if repeated exposure has a cumulative impact on skin properties (11). For instance, *N*-acetylglucosamine, commonly found in skin care products, was recently implicated as a driver of the skin microbiome (12). This compound is a precursor to hyaluronic acid, a major dermal and epidermal constituent whose biosynthetic pathway responds to UV irradiation (13), the major driver of accelerated skin aging. The investigation of microbiome composition determinants is critical to understanding whether specific factors contribute to a healthy skin microbial community or whether they indirectly influence host skin health via microbiome alterations.

Here, we examined poorly understood relationships between the skin microbiome composition and both intrinsic and extrinsic factors. We conducted a cross-sectional, walk-in, voluntary study convened at the 2015 ArtPrize Festival in Grand Rapids, MI. We collected forehead, nose crease, scalp, forearm, and oral epithelium samples from 495 subjects and characterized their bacterial microbiomes via sequencing of 16S rRNA genes (V4 region). We also compiled extensive subject metadata through standardized questionnaires and noninvasive biophysical and imaging methods. Our data analysis focused on (i) determining how skin microbiome variation is associated with demographic, lifestyle, and host physiologic factors and (ii) identifying specific microbiome signatures associated with chronological age and skin aging.

## RESULTS

### Study population.

Subjects (*n *=* *495) were both female (58%) and male (41%), had an average age of 40 years (range:9 to 78), and were primarily Caucasian (66%) or African-American (24%); the remaining 10% self-identified as either Hispanic, Asian, Native American, or mixed ethnicity (see Table S1A in the supplemental material). Over 90% of the subjects lived in the Grand Rapids metropolitan area. For each of the study participants, we compiled a data set consisting of 304 variables and microbial profiles. We considered 39 variables (Table S1B) for further study that (i) were previously shown or hypothesized to influence skin microbiome composition (10, 14–16) or (ii) may be affected by microbial composition or metabolism.

10.1128/mBio.00839-19.3 TABLE S1 Subject information and associations. (A) Demographic characteristics of ArtPrize participants. (B) Subject variables used for microbiome modeling. (C) Genera significantly associated with subject variables across all sites. (D) OTUs on forehead significantly associated with subject variables. (E) OTUs in mouth significantly associated with subject variables. (F) OTUs on nose significantly associated with subject variables. (G) OTUs on scalp significantly associated with subject variables. (H) OTUs on forearm significantly associated with subject variables. Download Table S1, XLSX file, 0.04 MB. Copyright © 2019 Dimitriu et al. 2019 Dimitriu et al. This content is distributed under the terms of the Creative Commons Attribution 4.0 International license.

### Characterizing the ArtPrize microbiome.

After removing samples with fewer than 1,000 sequence reads, we obtained microbiome data for 1,977 samples from five body sites representing distinct environments. While the V4 hypervariable region has been shown to cover most of the described human bacterial diversity, it is known that V4-specific primers are biased against some abundant skin bacteria, particularly *Propionibacterium* (17). However, V4 primers can detect taxa that are underrepresented in skin microbiome surveys using the V1-V3 region (18) and produce amplicons with lower error rates (19).

**FIG 1:**
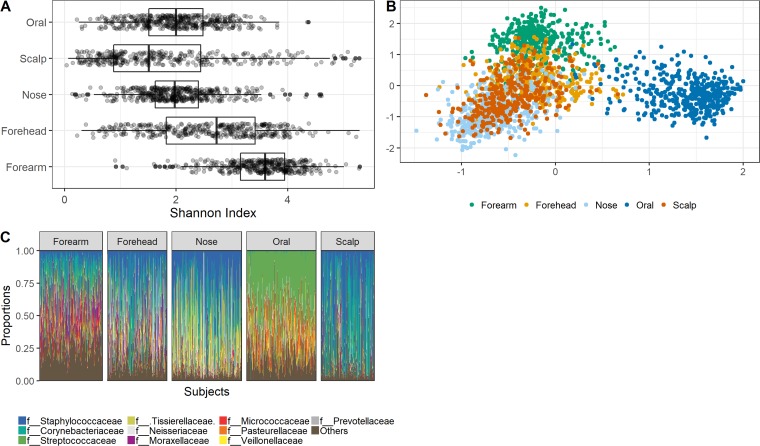
Microbiome diversity. (A) Bacterial alpha diversity at each site. (B) NMDS ordination displaying bacterial composition similarity (Bray-Curtis dissimilarities) among samples, color-coded according to site. (C) Stacked bar plot showing the relative abundance of the top ten most abundant bacterial families in each of the five sampled body sites. The “Others” category represents less-abundant taxa.

Alpha diversity (as estimated by the Shannon index) of microbiomes differed among sites, with the scalp showing the lowest diversity and the forearm, a dry site, the highest (Fig. 1A). Exploratory nonmetric multidimensional scaling (NMDS) visualization of Bray-Curtis dissimilarities confirmed earlier reports that body site is a major driver of microbiome composition (Fig. 1B). Interindividual variation in microbiome composition, which caused the microbiomes of distinct sites to overlap, mainly resulted from differences in the relative abundance of operational taxonomic units (OTUs) that were prevalent (in >50% of samples). Prevalent OTUs were primarily members of the families, *Staphylococcaceae*, *Corynebacteriaceae*, and *Streptococcaceae* (Fig. 1C), consistent with previous studies.

### Multivariate modeling of microbiome-host factor associations.

**FIG 2:**
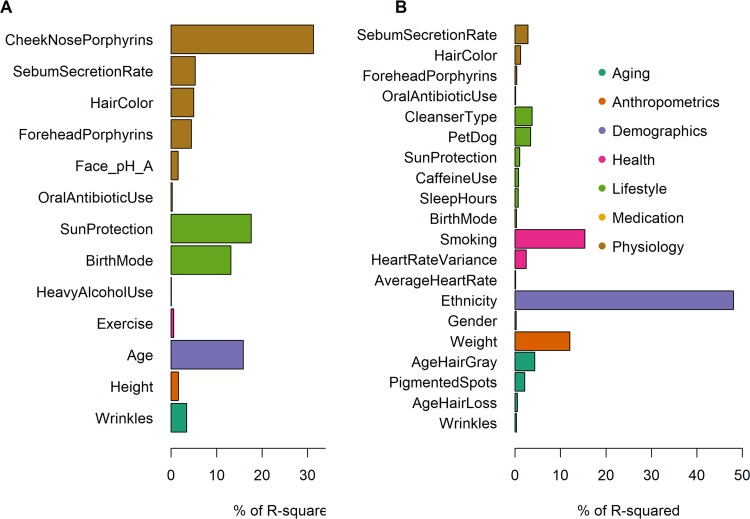
Effect sizes of variables on microbiome composition. Variables found to be significantly correlated with overall forehead (A) and oral (B) microbiome variation, sorted by their relative importance (% of *R*^2^) within predefined categories. *R*^2^ values represent the fractions of microbial composition variation explained by the variables in each category. Variables are additionally described in Table S1B.

We tested 39 variables (Table S1B) representing seven categories (host physiology, medication, lifestyle, health, demographics, anthropometrics, and skin aging), to identify factors associated with microbial community composition. First, we fit linear models onto ordination scores to determine which variables correlate with overall microbiome community variation at each site. Between 13 and 25 factors were found to correlate with microbial community composition (false discovery rate [FDR] < 10%), collectively explaining 11% (scalp), 14% (forearm), 15% (forehead), 16% (mouth), and 20% (nose) of the variation (distance-based redundancy analysis) (Fig. 2; see also Fig. S1 in the supplemental material).

10.1128/mBio.00839-19.1 FIG S1 Effect sizes of variables on microbiome composition. Variables found to be significantly correlated with overall nose (A), scalp (B), and forearm (C) microbiome variation. % *R*^2^ values represent the fractions of microbial composition variation explained by the variables in each category. Variables are additionally described in Table S1B. Download FIG S1, EPS file, 0.2 MB. Copyright © 2019 Dimitriu et al. 2019 Dimitriu et al. This content is distributed under the terms of the Creative Commons Attribution 4.0 International license.

To assess the relative importance of individual factors as predictors of community composition, we performed a multiple regression on Bray-Curtis dissimilarities and partitioned the explained variability (*R*^2^) attributable to each predictor (relative importance analysis). To avoid collinearity issues, we removed the covariates(s) that could be predicted from the remaining predictors with an adjusted *R*^2^ threshold of 0.8 with, at most, two variables filtered out from any site (redundancy analysis).

**FIG 3:**
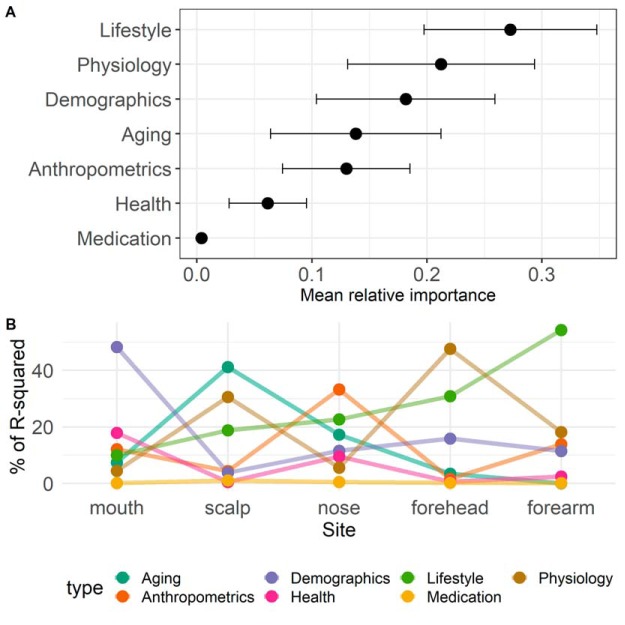
(A) Mean relative importance of variable categories. (B) Aggregate relative importance of categories stratified by site.

The contributing variables were different and had varying explanatory power across sites. Forehead porphyrins and cheek porphyrins, the use of skin protection, ethnicity, age, height, and hyperpigmented spots were among the strongest predictors of community composition on skin sites (Fig. 2A; Fig. S1). In the mouth, smoking (determined as the number of years smoked × number of packs) and ethnicity were the dominant host factor shaping microbiome composition (Fig. 2B). When averaged across all sites, the top three metadata categories contributing to microbiome variation were lifestyle, physiology, and demographics (Fig. 3A). However, the aggregate relative importance of categories differed greatly across sites. For instance, where the importance of lifestyle was low, the importance of skin aging was high and vice versa (Fig. 3B).

### Identification of OTU-host factor associations.

**FIG 4:**
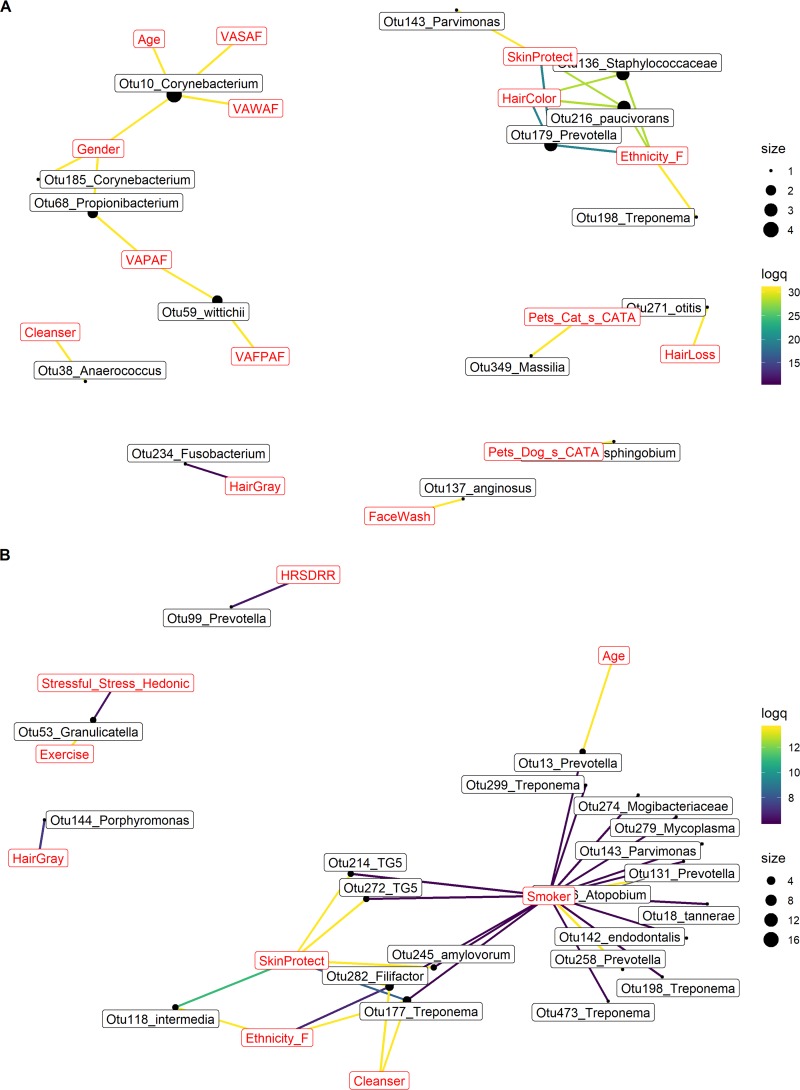
Associations between bacterial taxa and subject variable categories. Networks displaying the top associations between OTUs and variable categories on the forehead (A) and in the mouth (B). Edges are color-coded according to the log-transformed permutation test q-values (see HAllA methods for additional details); yellow hues indicate stronger associations. Node size is proportional to the number of associations between OTUs and variables. Variable codes are explained in Table S1B and the main text.

Next, we performed a hierarchical all-against-all analysis (HAllA [http://huttenhower.sph.harvard.edu/halla]; G. Rahnavard, E. A. Franzosa, L. J. McIver, E. Schwager, J. Lloyd-Price, G. Weingart, Y. Sup Moon, X. C. Morgan, L. Waldron, and C. Huttenhower, unpublished data) to study associations between individual OTUs and covariates. By relying on mutual information between variables, HAllA is ideally suited for the identification of robust associations between high-dimensional data sets containing both continuous and categorical variables. To visually ascertain the overall strength of metadata-taxa associations at each body site, we (i) retained the 80th percentile of log-transformed q-values for each association pair and (ii) counted the number of OTUs associated with each covariate (Fig. 4). We enhanced visualization-based interpretation of statistically significant associations by considering all taxa significantly associated with covariates (Table S1C to H).

In total, 67 genera were found to be associated with at least one host factor at a minimum of one site (Table S1C). In general, the strongest associations involved aging, demographics, and lifestyle factors, with members of the genera *Anaerococcus*, *Peptoniphilus*, *Prevotella*, and *Corynebacterium* dominating these associations (Table S1C). On the forehead, *Corynebacterium* OTUs were associated with chronological age and skin aging (hyperpigmented spots [VASAF] and wrinkles [VAWAF]), while a *Propionibacterium* OTU (OTU68) was associated with forehead porphyrins (VAFPAF) (Fig. 4A; Table S1D). In the mouth, the most connected genera—*Prevotella*, *Treponema*, and *Leptotrichia*—were associated with smoking and demographics (ethnicity) (Fig. 4B; Table S1E).

The three genera with the most associations on the nose (*Prevotella*, *Anaerococcus*, and *Corynebacteria*) were associated with physiology (cheek porphyrins [VAPAF]), lifestyle (use of facial cleanser), and aging (age at which hair turned gray) (Fig. S2A and Table S1F). On the scalp, associations were relatively sparse, and largely involved demographic factors (Fig. S2B and Table S1G). There, *Staphylococcus*, *Anaerococcus*, and *Corynebacterium* were strongly associated with ethnicity and porphyrins. Finally, several forearm OTUs, mostly affiliated with *Chryseobacterium*, *Neisseriaceae*, and *Corynebacterium*, were found to be associated with physiological, demographic, and lifestyle factors (Fig. S2C and Table S1H). Using this integrative approach, we confirmed the broad importance of host factor categories revealed by multivariate modeling.

10.1128/mBio.00839-19.2 FIG S2 Associations between bacterial taxa and subject variables. Networks displaying the top associations between variables and (A) nose OTUs, (B) scalp OTUs, and (C) forearm OTUs. Edges are color-coded according to the log-transformed permutation test q-values (see HAllA methods for additional details); yellow hues indicate stronger associations. Node size is proportional to the number of associations between OTUs and variables. Download FIG S2, EPS file, 0.3 MB. Copyright © 2019 Dimitriu et al. 2019 Dimitriu et al. This content is distributed under the terms of the Creative Commons Attribution 4.0 International license.

### Forehead microbiome-based modeling of age.

**FIG 5:**
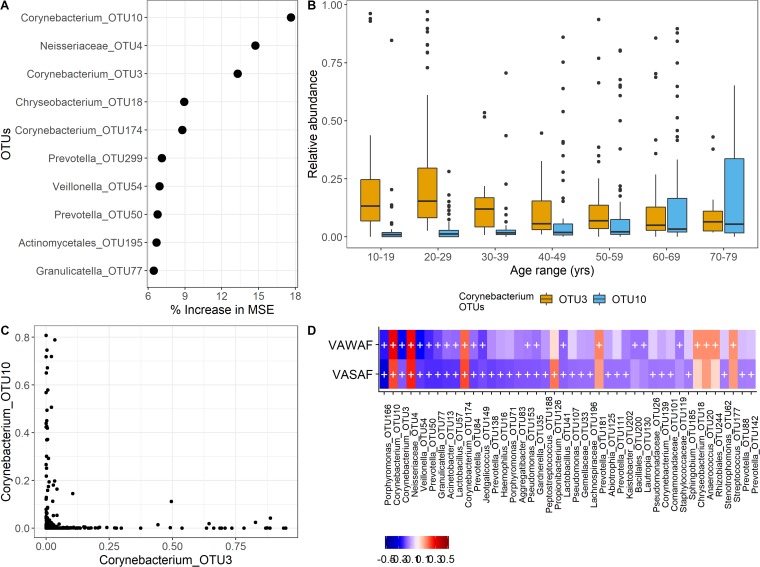
Forehead microbiome and age. (A) Relative importance of OTUs explaining age as a function of their contribution to the Random Forest model mean square error (MSE). (B) Subject age in relation to the relative abundance of *Corynebacterium* OTUs deemed most important in the Random Forest age model. (C) Relative abundance-based coexclusion of the age-predictive corynebacterial OTUs. (D) Spearman correlations between forehead OTUs (those present in at least 25% of the samples) and the skin aging variables Wrinkles (VAWAF) and PigmentedSpots (VASAF). The color bar represents correlation values, and the crosses represent significant associations (FDR < 0.05).

To more extensively probe the relationship between age and the microbiome, we performed a Random Forest analysis to predict host age based on forehead microbial features. With this model, we explained 32% of the variability in age and found that two *Corynebacterium* OTUs were among the three taxa with the highest relative importance (Fig. 5A). Interestingly, one of these (OTU10) tended to displace another (OTU3) in middle age, most frequently 40 to 49 years (Fig. 5B), in accordance with their high degree of coexclusion (Fig. 5C).

We performed a Spearman correlation analysis of two skin aging features that correlate with chronological age, wrinkles and hyperpigmented spots. We found that numerous forehead OTUs, including OTU3 and OTU10, were positively or negatively associated with wrinkles or spots (Fig. 5D).

**FIG 6:**
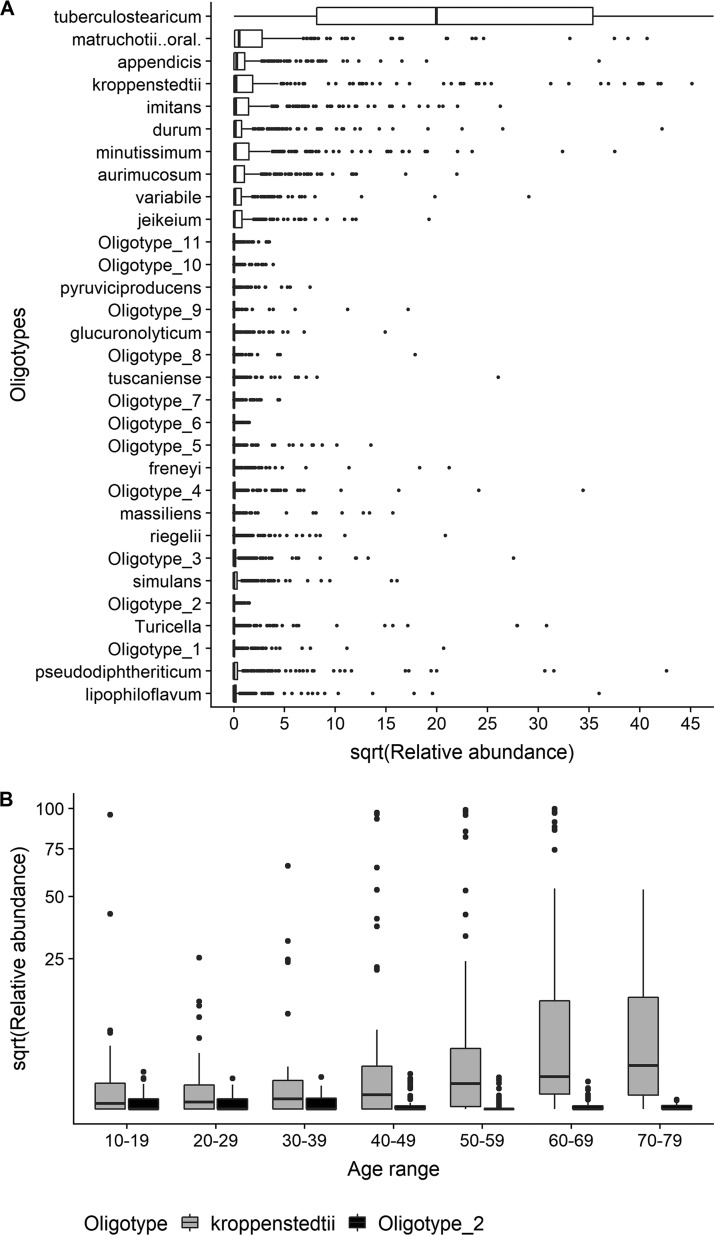
Oligotyping analysis. (A) Distribution of *Corynebacterium* oligotypes, sorted by overall median relative abundance. (B) Relative abundance among age classes of oligotypes tentatively assigned to corynebacterial OTU3 and OTU10.

To determine whether OTU3 and OTU10 on the forehead could be taxonomically classified below the genus level, we performed an oligotyping analysis (20) to resolve sub-genus-level sequence variants. We identified 31 *Corynebacterium* oligotypes, of which 20 could be assigned to species (BLAST similarity score = 100%; Fig. 6A). Only one oligotype, identified as *C. kroppenstedtii*, had a similar trend as OTU10, while only one oligotype, not assigned to a species, had a similar trend as OTU3 (Fig. 6B).

## DISCUSSION

We conducted a cross-sectional study aimed at better understanding the role of intrinsic and extrinsic host factors in shaping the skin microbiome. Our study is unique because it combines modern methods in microbiome research with classical noninvasive methods of cosmetic and dermatologic science. We found that demographic, lifestyle, and physiological factors were most associated with skin microbiome composition, while the influence of health parameters was strongest in the mouth. The concurrent analysis of several sites on the same subjects indicated the relative importance of host variables interacting with the microbiome. Our results suggest that two well-described properties of the skin microbiome, biogeographical specificity and temporal stability (8), are not only the result of host selection but are also influenced by extrinsic factors.

Our findings resemble what has been observed in the gut, although the specific factors linked to the intestinal microbiome are either unique to the gut or are ranked differently in terms of their relative importance (21, 22). This reflects the ecological uniqueness of body sites, and indeed we found site-specific microbial-covariate associations. For instance, at high-diversity, environmentally exposed sites, the contribution of lifestyle factors to microbiome variation tended to be high. Bacterial interaction networks of sites subject to environmental exposure show higher connectedness than those of occluded sites (23). We suggest that exposed skin sites harbor diverse microbiomes that are potentially more robust to community disruption by environmental fluctuations.

We also identified taxon- and community-level associations with factors that have not been well characterized in relation to the skin and oral microbiomes. The amount of porphyrins, a physiological parameter, was associated with microbial taxa at most skin sites. Porphyrins are a group of proinflammatory metabolites significant in acne development (24). It is understood that Propionibacterium acnes plays an important role in the synthesis of porphyrins on skin (25). Our results indicate that other microbes not known to do so may also participate in the production of porphyrins or modulate porphyrin production by Propionibacterium acnes. It is alternatively possible that porphyrin levels (determined by the activity of specific P. acnes strains) directly affect the presence of other bacteria.

Our integrated analyses highlight ethnicity as an important demographic variable interacting with the skin and oral microbiomes. On the forehead, HAllA identified hair color, the use of sun protection, and ethnicity as all correlated to a cluster of taxa dominated by *Clostridiales* genera (*Anaerococcus*, and *Peptoniphilus*). These taxa have been found to be enriched on the skin of patients suffering from primary immunodeficiencies (26), consistent with their role as opportunistic pathogens (27). In the mouth, a diverse group of taxa, including *Prevotella*, *Treponema*, and *Leptotrichia*, was likewise found to be associated with ethnicity and hair color. Heritable genetic variation is a component of ethnicity and therefore a possible contributor to microbiome-ethnicity relationships (28). Another possibility is that taxa–hair color–ethnicity links are indirectly driven by microbial interactions with melanocytes, melanin-producing cells whose metabolic activity changes in response to UV exposure and stimuli such as hormones, proinflammatory molecules, or injury (29).

Age groups spanning various stages of skin aging are not well represented in the skin microbiome literature. Among Finnish children (≤14 years old), age is associated with an increase in the abundance of *Actinobacteria* and *Proteobacteria* at the expense of *Lactobacillales* (15). Chinese individuals, on the other hand, harbor a progressively lower abundance of *Firmicutes*, Staphylococcus aureus in particular, as they approach old age (30). The foreheads of older healthy Japanese women (>60 years old) are depleted in *Propionibacterium* (31). We similarly identified species-specific signatures in the forehead that may reflect physiological aging processes. In our study, age was associated with two corynebacterial taxa that mutually coexclude each other. While coexclusion dynamics, or “dust bunny distributions,” can be caused by several mechanisms, dust bunnies are mostly influenced by the number of environmental gradients (32). Chronological age likely integrates many underlying environmental gradients, including intrinsic and extrinsic aging processes.

What drives the dominance of *C. kroppenstedtii* after middle age? One possibility is that *C. kroppenstedtii* specifically metabolizes fatty acids typically found on skin at old age (33). Although considered a skin commensal, there is evidence that this species can act as an opportunistic pathogen (34). The case of *C. amycolatum* may offer clues about the occurrence of *C. kroppenstedtii*. *C. amycolatum* mediates the induction of the antimicrobial protein RNase 7 (35), and like *C. kroppenstedtii*, lacks mycolic acids. Elderly individuals have an increased susceptibility to infections, partly because of a suppressed antimicrobial peptide (AMP) landscape, which suggests a dampened ability of aging skin to control growth of specific corynebacteria. It remains to be seen whether this checkpoint only pertains to nonmycolic acid-producing *Corynebacterium* species. It may be that modulation of AMP expression is strain specific. Cultivation may therefore reveal whether distinct strains elicit differential host responses, as well as provide genomic bases behind those responses.

The relationship between age and the two *Corynebacterium* OTUs was similar across skin sites, although it was absent in the mouth (data not shown). These observations suggest a link between the skin microbiome and age that ranges from episodic (in the forearm, for instance) to potentially predictive, as we have seen on the forehead. It is possible that the various degrees of connectedness between the microbiome and age depends on the strength of the link between skin aging (e.g., appearance of wrinkles and spots) and specific microbial signatures. In the forehead at least, taxa associated with chronological age were also related to skin aging. While skin aging is partly dictated by lifestyle (36), the lifestyle factors we measured did not affect taxa associated with skin aging, suggesting that certain species are selected by the host independently of external perturbations (8). Further studies that collect relevant lifestyle data (for example, a detailed record of skin care practices and diet) are needed to determine whether certain host habits affect skin aging directly or via the involvement of microbial components. With this information, we will be able to assess whether skin aging can be modulated by manipulating the microbiome.

In the gut, medication has been shown to correlate strongly with the gut microbiome composition of “average” Western populations (21). Topical and oral antibiotic exposure (measured as the time since the medication was last taken) did not play a significant role in shaping skin or oral microbiome composition in our cohort, in agreement with other recent studies (14, 37). This finding may reflect the coarseness of our survey, in which antibiotic use was not stratified by type, underlying condition, or route of delivery. Duration of antibiotic exposure, which we did not record, is also likely to affect the microbiome. A further possibility is that skin microbiome responses to antibiotic perturbations are highly individualized, as is the case in the gut (38).

Similar to other recent studies (21, 22, 39), our multivariate models explained 12 to 20% of the nonredundant variability in the microbiome. There are several explanations for these relatively low effect sizes, which were likely the result of inherent study limitations. First, the level of host phenotypic detail that we derived from our questionnaire was limited. For instance, we did not record the subjects’ allergy phenotype or allergy-related factors, including length of breastfeeding and interaction with animals other than pets. Moreover, we did not consider important clinical information such as history of disease and medication, which may be particularly relevant to the oral microbiome (40). Second, although the physiological parameters provided a comprehensive picture of host parameters that may either be modulated by, or modulate, microbial activity, extensive profiling of the skin’s chemical milieu is likely to explain more of the variance in microbiome composition. Third, because of the large interindividual variability, driven in part by a transient pool of microbial colonizers shared among individuals, pets, and homes (41), the proportion of microbiome compositional variation that can be explained is inherently limited.

Our study has two other important limitations. One is its reliance on participants’ retrospective self-reported data of their lifestyle and health. To mitigate potential biases due to information collected with questionnaires, we focused on (i) covariate types that were previously found in gut-focused studies to be associated with microbiome features (21, 42) and (ii) covariates deemed to be broadly representative of metadata categories. Another limitation is primer bias. It is known that amplification of the V4 region of the 16S rRNA gene is biased against Propionibacterium acnes (43). Our study, however, was designed to obtain a general overview of microbiome variation, as in recent studies (30, 37). Compared to the V1-V3 primer pair, V4 primers better detect *Finegoldia* and *Peptoniphilus* (18). These taxa, and others in the *Peptostreptococcacea* family, may be involved in important host-microbe interactions; for example, they are affected by the use of skin products (44) and typify dysbiotic states of immunocompromised individuals (26).

In conclusion, building upon a comprehensive metadata set of a representative Mid-Western population, we identified a group of variables which in combination explained up to 20% of the variation in skin microbiome composition. This suggests the influence of additional, currently unmeasured covariates, as well as intrinsic ecological processes such as founder effects, interspecies interactions, and historical contingencies. We showed that some of the variables identified as relevant to microbiome variation are currently understudied and should be considered in future cohort studies. Finally, although our study focused on a North American population, many of our findings are likely true of other populations.

## MATERIALS AND METHODS

### Study design.

The study was a single-center, single-visit cross-sectional study. The study took place in October of 2015 in downtown Grand Rapids, MI, during the 2-week period of the 2015 ArtPrize art festival. Participants were eligible if they were over 10 years of age, were not pregnant or lactating, and did not wear wigs or toupees. An IRB-approved informed consent form, consistent with the requirements in 21 Code of Federal Regulations (CFR) 50.25, was used to obtain consent from every subject. After enrollment, subjects proceeded through a series of 10 stations in the following order: microbiome swabbing, head hair imaging, skin surface pH, facial cleansing, electronic questionnaires, heart rate variance, facial imaging, hair clipping, skin elasticity, and sebum secretion rate.

### Skin, scalp, and oral microbiome sampling.

Skin, scalp, and oral samples were collected in the following sequence: (i) left ventral forearm, (ii) left cheek-nose crevice, (iii) central forehead, (iv) midline of the scalp vertex and (v) left and right buccal surfaces. Sterile Catch-All sample collection swabs were used for all sites. For skin and scalp sites, the swabs were wetted with sterile buffer (50 mM Tris, 1 mM EDTA, 0.5% Tween 20 [pH 7.6]). The skin was pulled taut and rubbed with the swab for 30 s with firm pressure. For the buccal mucosa surfaces, the swab was used without wetting and gently rubbed on the left and right inner cheek surfaces. Swab heads were clipped, placed in PowerSoil DNA isolation kit (Mo Bio Laboratories, Inc., Indiana) lysis buffer, and stored temporarily at –20°C for 24 h and then at –80°C until microbiome analysis (45).

### Subject metadata.

The self-administered electronic questionnaire gathered demographic, health, lifestyle, medication, and physiological information on each subject. Specific variables used for microbiome modeling, and their definitions (i.e., the answers that were provided to each question) are shown in Table S1B.

### Skin surface pH.

Skin pH measurements were made on the left forearm (midway between the elbow and the wrist) and the left cheek (about 3 to 4 cm below the left corner of the eye). Measurements were made with a Mettler-Toledo Seven2Go8 Pro equipped with an InLab surface flat-surface electrode (Mettler-Toledo, Columbus, OH) and calibrated twice a day with pH 4 and pH 7 standard buffer, as previously described (46).

### Heart rate variance.

Heart rate variance (HRV) was measured using the PowerLab 16/35 data acquisition system (AD Instruments, Colorado Springs, CO) equipped with a photoplethysmographer and LabChart Pro software. Subjects were seated comfortably in a quiet area, and the finger probe was strapped to the ventral skin pad of the middle distal phalange. Subjects closed their eyes, and measurements were collected over a 5-min period. The average heart rate and the standard deviation of the R-R intervals was calculated using the HRV toolkit in the LabChart Pro software and used in statistical analysis.

### Facial imaging and image analysis.

Facial images (left and right oblique view) were captured using the VISIA-CR 4.1 (Canfield Scientific, Fairfield, NJ) equipped with a Canon EOS-5Ds Mk III SLR camera. Images were analyzed for severity of facial features (wrinkles, hyperpigmented spots, red features, and porphyrins) in selected regions of interest (periorbital, forehead, and cheek regions) using a Vaestro image analysis toolkit (Canfield Scientific). Facial features were expressed in terms of area fractions.

### Sebum secretion rate.

The amount of sebum secreted from the forehead in a fixed amount of time was measured via image analysis of Sebutapes (CuDerm, Dallas, TX). Briefly, the subject’s facial forehead skin was cleansed with facial cleanser (Cetaphil daily facial cleanser) and then wiped with the isopropanol cotton wipes to remove any remaining sebum. Two Sebutape patches were adhered to the forehead symmetrically around the midline of the face for between 45 and 90 min. The Sebutape patches were removed, and the area of translucency was quantified. Sebum secretion rate in area/min was calculated by dividing the area of translucency by the time the patch was on the forehead.

### DNA extraction, amplicon sequencing, and sequence processing.

DNA from clipped swabs was extracted with the PowerSoil DNA isolation kit according to the manufacturer’s instructions. Bacterial 16S rRNA genes were amplified with a primer set (515F/806R) targeting the V4 region using a dual-barcoding approach (19) in 50-μl reaction mixtures containing 1× Phusion Hot Start II DNA polymerase (Thermo Fisher Scientific). Amplification conditions were as follows: 98°C for 2 min, followed by 30 cycles of 98°C for 20 s, 55°C for 15 s, and 72°C for 30 s and then a final 10-min extension at 72°C. For each sample, we amplified a single replicate. Amplicons were purified and normalized using a SequalPrep kit (Invitrogen, Eugene, OR). Purified amplicons were quantitated with a Qubit 2.0 fluorometer and pooled for Illumina sequencing. Sequencing was performed on an Illumina MiSeq system using a 2 × 250-bp paired-end version 2 MiSeq reagent kit. To assess the impact of contamination, we included a swab, an extraction kit-only, and a DNA template-free control in each 96-well plate. Except for the template-free controls, all controls were processed in the same way as the skin and mouth swabs. An OTU was flagged as a potential contaminant if its mean average abundance in controls exceeded 25% of the average abundance in skin samples.

Raw sequencing reads were curated with mothur (v1.38.1) (47). After merging reads under default settings, sequences with long homopolymers (*n* = 8) were removed and aligned against a SILVA-based reference alignment (48) using a profile-based aligner (49). Aligned sequences were preclustered using a 2-nucleotide threshold (50). *De novo* chimera detection was done with the abundance-based algorithm implemented in UCHIME (51). Denoised reads were clustered into OTUs at a 97% sequence identity threshold using the average neighbor algorithm and classified with the Wang method (52) against the Greengenes database (v. 13_8_99).

### Metadata curation.

Host measurements and questionnaire data were manually curated to be representative of categories relevant to skin health and aging. Missing values in the preselected variables were imputed with the missForest R package under default settings. Variables with more than 20% missingness were deleted. For multivariate modeling of metadata-microbiome associations, we determined how well each variable can be predicted from the remaining variables using parametric additive models (*redun* function of the R Hmisc package). A variable was deemed redundant when the other variables predicted it with an *R*^2^ of 0.8 (adjusted for the number of predictors) and was removed before analysis.

### Metadata-microbiome associations.

To examine the importance of host variables on overall microbiome composition, we calculated the association between continuous or categorical metadata and community ordinations for each site (NMDS based on Bray-Curtis dissimilarities) with the *envfit* function of the vegan package (v.2.4) (10,000 permutations; FDR < 10%). The variables identified as significant by *envfit* were used in a distance-based redundancy analysis to estimate their combined explanatory power on microbiome variation (*dbrda* function, vegan package). We then performed a multiple regression on log-transformed Bray-Curtis dissimilarities to assess the relative contribution of individual predictors to microbiome variation. To that end, we first computed distances from covariates (Gower for nonnumeric and Euclidean for numeric ones) and then used a relative importance metric, *lmg*, which partitions the adjusted-*R*^2^ by averaging over orders among predictors (*calc.relimp* function, relaimpo R package).

Finally, we determined (i) whether specific taxa drive the associations between microbial community composition and subject variables and (ii) the broad-level factors (i.e., the metadata categories) affecting skin microbiome variation at the OTU level. To that end, we used hierarchical all-against-all association testing (HAllA, v.0.6.14) under the following settings: q-value threshold, 0.05; measure of association, normalized mutual information; and Benjamini-Hochberg FDR correction. We only considered OTUs present in at least 25% of the samples. OTU counts were Hellinger-transformed to downweigh the influence of sparse taxa prior to analysis.

### Microbiome-age modeling.

We used Random Forests using default parameters to model taxa-age associations [*randomForest* R package; (53)]. Model performance was estimated using the out-of-bag (OOB) mean of squared residuals, an unbiased estimate of generalization error for regression (54), and percent variance explained. Experiments comparing OOB and test sample error estimates show only small differences between them (54).

### Oligotyping analysis.

We used oligotyping to identify species-level variation in the genus *Corynebacterium.* The minimum substantive abundance threshold for an oligotype (-M) was set to 500 reads, and the minimum number of samples (-s) and percent abundance cutoff (-a) were set to 500 and 5%, respectively. To map oligotypes to OTUs, we compared the relative abundance of the most abundant corynebacterial OTUs (i.e., 3 and 10) across age groups to the relative abundance of all oligotypes. Oligotypes were deemed as species if they had a BLAST similarity score of 100% to their closest matches.

### Data availability.

Raw sequencing reads are available through the NCBI Short Read Archive under BioProject accession PRJNA542898. The materials necessary to reproduce the Random Forest analysis are available at figshare (10.6084/m9.figshare.8127287).
